# Malaria chemoprophylaxis and the serologic response to measles and diphtheria-tetanus-whole-cell pertussis vaccines

**DOI:** 10.1186/1475-2875-4-53

**Published:** 2005-11-06

**Authors:** Jennifer B Rosen, Joel G Breman, Charles R Manclark, Bruce D Meade, William E Collins, Hans O Lobel, Pierre Saliou, Jacquelin M Roberts, Pierre Campaoré, Mark A Miller

**Affiliations:** 1 Division of International Epidemiology and Population Studies, Fogarty International Center, National Institutes of Health, Bethesda, MD 20892, USA; 2 Howard Hughes Medical Institute-National Institutes of Health Research Program, Bethesda, MD 20892, USA; 3 Division of Bacterial Products, Allergenic and Parasitic Products, Center for Biologics Evaluation and Research, Food and Drug Administration, Bethesda, MD 20892, USA; 4 Division of Parasitic Diseases, National Center for Infectious Diseases, Centers for Disease Control and Prevention, Atlanta, GA 30333, USA; 5 Société de Pathologie Exotique, Paris, France; 6 01 BP 1587 Ouagadougou 01, Burkina Faso

## Abstract

**Background:**

Acute malaria has been associated with a decreased antibody response to tetanus and diphtheria toxoids, meningococcal, salmonella, and Hib vaccines. Interest in giving malaria drug therapy and prevention at the time of childhood immunizations has increased greatly following recent trials of intermittent preventive therapy during infancy (IPTi), stimulating this re-analysis of unpublished data. The effect of malaria chemoprophylaxis on vaccine response was studied following administration of measles vaccines and diphtheria-tetanus-whole cell pertussis (DTP) vaccines.

**Methods:**

In 1975, six villages divided into two groups of children ≤74 months of age from Burkina Faso, were assigned to receive amodiaquine hydrochloride chemoprophylaxis (CH+) every two weeks for seven months or no chemoprophylaxis (CH-). After five months, children in each group received either one dose of measles or two doses of DTP vaccines.

**Results:**

For recipients of the measles vaccine, the seroconversion rates in CH+ and CH- children, respectively, were 93% and 96% (P > 0.05). The seroresponse rates in CH+ and CH- children respectively, were 73% and 86% for diphtheria (P > 0.05) and 77% and 91% for tetanus toxoid (P > 0.05). In a subset analysis, in which only children who strictly adhered to chemoprophylaxis criteria were included, there were, likewise, no significant differences in seroconversion or seroresponse for measles, diphtheria, or tetanus vaccines (P > 0.05). While analysis for pertussis showed a 43% (CH+) and 67% (CH-) response (P < 0.05), analyses using logistic regression to control for sex, age, chemoprophylaxis, weight-for-height Z-score, and pre-vaccination geometric mean titer (GMT), demonstrated that chemoprophylaxis was not associated with a significantly different conversion rate following DTP and measles vaccines. Seven months of chemoprophylaxis decreased significantly the malaria IFA and ELISA GMTs in the CH+ group.

**Conclusion:**

Malaria chemoprophylaxis prior to vaccination in malaria endemic settings did not improve or impair immunogenicity of DTP and measles vaccines. This is the first human study to look at the association between malaria chemoprophylaxis and the serologic response to whole-cell pertussis vaccine.

## Background

Malaria accounts for an estimated 1 to 3 million deaths each year, with the majority occurring in children under five years of age in sub-Saharan Africa [1]. Vaccine-preventable diseases cause an estimated 1 to 2 million deaths in African children [2]. The WHO's Expanded Program on Immunization (EPI) is targeted at malarious areas, emphasizing the need to understand the effect of malaria and antimalaria drug use on vaccine immunogenicity and efficacy. Accordingly, a study that began in 1975 has been fully analysed following great increasing recent interest in the important topic of malaria chemoprophylaxis and, in particular, intermittent preventive (malaria) therapy of infants (IPTi) [3-7].

Acute malaria has been associated with a decreased response to tetanus toxoids, and meningococcal polysaccharide, Hib conjugate, and whole cell vaccines for typhoid fever [8-10]. Asymptomatic parasitaemia has been associated with a decreased response to the newer acellular pertussis and meningococcal vaccines, suggesting a benefit from malaria prophylaxis prior to vaccination [11-13]. Other studies have shown that asymptomatic parasitaemia or anti-malarial drug administration does not inhibit vaccine response to various live, attenuated, whole-cell killed, and toxoid vaccines [4,5,14-20]. No human studies have looked at the association between parasitaemia and the serologic response to whole-cell pertussis vaccine, a product still used in many vaccination programmes, particularly in developing countries. Antimalarials may also depress vaccine response as illustrated by the immunodepressive effect of 4-aminoquinolones[13,21-24].

The study aimed to determine the effect of malaria chemoprophylaxis on vaccine seroconversion or seroresponse to live, attenuated measles vaccine, diphtheria and tetanus toxoids and whole-cell pertussis (DTP) vaccines.

## Methods

### Study area and population

The study was conducted from May through December in 1975 in six villages; all were located in the Guinean savanna and were hyper- and holo-endemic for malaria, depending on transmission season [25]. Before the study began (February-March, during the low transmission season), a 52% *Plasmodium falciparum *parasitaemia prevalence was found in 150 children (25 per site) <6 years of age, with no major differences between the sites; during this pre-study investigation, antibodies to *P. falciparum *were detected by indirect haemagglutination (IHA) in 100 percent of children tested from five of the six villages (25 children per village). Burkinabe clinicians in the nearest dispensaries and hospitals stated that the study area was endemic for measles (cases and deaths occurred during the study), diphtheria, tetanus, and pertussis, but the incidence was unknown; routine data had not been collected from the study villages because the EPI had not yet begun [26]. Hence, previous vaccination of children was extremely unlikely and was confirmed by interrogation of individual families. There was no malaria prophylaxis; treatment for fevers and other illness was obtained from traditional healers and in dispensaries within five km of the villages. *P. falciparum *resistance to 4-aminoquinolones was unknown in the area. Exclusion criteria for participation in the study included acute or chronic severe illness and the presence of detectable pre-vaccination measles antibodies. Children were weighed with a calibrated hanging scale. The length of very young children was measured with them lying down on a calibrated board and, for older children, standing, according to Centers for Disease Control and Prevention (CDC) nutrition programme guidelines.

### Study design

**Figure 1:**
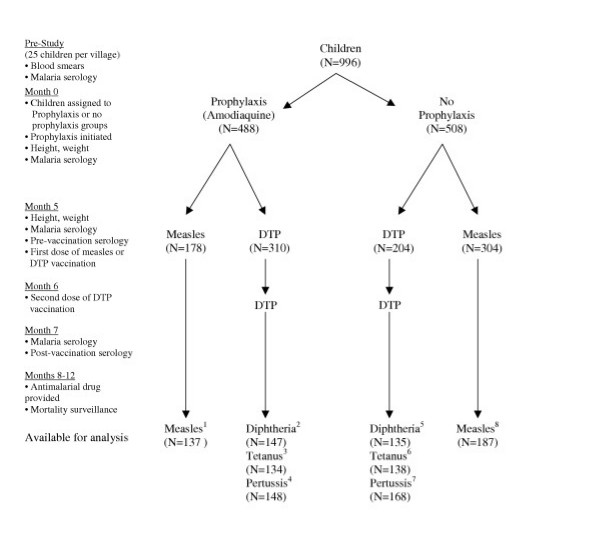
**Malaria chemoprophylaxis and response to childhood vaccinations: flow chart of study design**. Number of subjects excluded from analysis due to moving, death or lack of available sera: 1. 4 (37 excluded due to detectable pre-vaccination measles antibody titers) 2. 163 3. 177 4. 162 5. 70 6. 66 7. 36 8. 31 (68 excluded due to detectable pre-vaccination measles antibody titers, 18 excluded due to receipt of some prophylaxis)

Children, aged 4 to 74 months (N = 996) living in the 6 malarious villages were assigned to 2 groups of 3 villages each, depending on whether they lived in villages east or west of Bobo-Dioulasso. One group received amodiaquine prophylaxis, CH+ [N = 488], and the other received no prophylaxis, CH- [N = 508]. Virtually all children within the same village received either measles [N = 482], or DTP vaccine [N = 514] (Figure 1). The study villages had differing populations of target-aged children. Hence, village and subject separation into measles versus DTP groups was based on the estimated number of children needed for each vaccine, and was dependent on the initial calculation of sample size (see Statistical Analysis). There was no blinding of study participants or researchers.

The populations were of a related Mandinke ethnic group (Bobo in the eastern and Senufo in the western villages) and the communities had similar *Anopheles gambiae *ecology. Villages were chosen, based on the level of endemicity of malaria, the immunologic naivete of those receiving immunizations, and their proximity to one another; villages were spaced far enough apart so that families in CH- villages did not know that children in CH+ villages were receiving chemoprophylaxis, yet were close enough (within 75 km of Bob-Dioulasso) for travel convenience and management by the research team.

### Chemoprophylaxis

CH+ children received a single prophylactic dose of amodiaquine hydrochloride suspension or tablets every 2 weeks for 7 months beginning in May and June, the start of the transmission season; those <12 months of age received 100 mg, those 12 to 47 months received 200 mg, and those 48 to 73 months old received 300 mg. Both amodiaquine and chloroquine are 4-aminoquinolines. Amodiaquine is the prodrug for the active ingredient desethylamodiaquine (DAQ). DAQ has a terminal half life of one to three weeks and is schizonticidal in very low concentrations [27,28]. While amodiaquine lost favour because of its association with agranulocytosis, the drug is now being re-evaluated. Because amodiaquine has not been used for over 2 decades, it has somewhat greater efficacy than chloroquine for *P. falciparum *resistant to chloroquine [29,30].

Seroconversion rates were measured in all CH+ and CH- children for whom paired sera were available. In addition, a CH+ group having strict compliance to drug ingestion was analyzed to examine more carefully the effect of malaria or drug use on serconversion and seroresponse. For this study, criteria for strict compliance included: receipt of ≥75% of the doses, no two consecutive doses missing, no doses missed in the month prior to vaccination(s), and no doses missed between the second vaccination and the last blood draw. At study completion, during the beginning of the low transmission season, families of children in the CH+ group were given a short-term supply of amodiaquine prophylaxis and instructions for home treatment in case of a rebound malaria attack. Follow-up visits occurred every one to two months in all villages for six months after the study.

### Vaccination

All children were vaccinated with either measles vaccine or the first dose of DTP at month 5 (October or November, peak malaria transmission) and the second DTP dose one month later. Two doses of DTP were given during the study (rather than the standard initial series of three doses) to increase the likelihood that any effect from chemoprophylaxis would be discerned; a third dose was given at the end of the study but bloods were not drawn after this third vaccination. Vaccinations and amodiaquine were administered on the same day. The manufacturer and source of the licensed vaccines used were the Dow, Lirugen (Schwarz strain) measles vaccine, Lot No. 185806 AA and Merrill-National DTP, adsorbed, USP, vaccine, filling number 1036 DM, bulk Lot No. 1832. Measles and DTP vaccines were injected intramuscularly (0.5 ml) via a hydraulic (pressurized), injection device, the Ped-O-Jet^® ^injector. Following study completion, all children in participating villages received the vaccine that they did not receive during the study. Prior to use, the measles and DTP vaccines were tested for potency and met standard requirements.

### Vaccine serology

Venous blood samples were kept refrigerated and within 1 to 3 days serum was separated and kept at -20°C prior to shipment to the CDC, Atlanta, Georgia, USA, in dry ice. Serologic testing for the measles vaccine was performed in 1977 at the then Virology Division, Bureau of Laboratories, CDC. Antibody response to the DTP vaccine was performed in 1977 at the then Division of Bacterial Products, Bureau of Biologics, Food and Drug Administration, in Bethesda, MD.

Measles antibody was assessed by haemagglutination inhibition [31-33]. Seroconversion was defined by a rise in titer to ≥1:20 from an initial titer of <1:10 (the lowest detectable titer). Diphtheria and tetanus antibody titers were measured by passive microhemagglutination, using tanned sheep red blood cells [34,35]. Individuals showing a >4-fold rise in antibodies to uncoated sheep red blood cells were not included in the analysis. Pertussis titers were measured by microagglutination using killed cells of *Bordetella pertussis *strains 134 and 165 [36]. The titer is the log_2 _of the reciprocal of the highest final serum dilution resulting in detectable agglutination. When sufficient serum was available, the lowest final serum dilution tested was 1:8; by convention, samples negative at a 1:8 dilution were assigned the titer log_2 _= 2. If the serum sample volume was low, higher initial dilutions were used. When such sera were negative at the lowest dilution tested, the titer was reported as <lowest dilution tested. Because the actual end-point was not known, titers for these sera were not included in the calculation of geometric mean titer (GMT) or geometric mean fold rise in titer (GMR). For some individuals, it was possible to verify a ≥4-fold rise even if the actual endpoint was not known for both sera. A ≥4-fold rise for DTP antigens was considered positive. For DTP, in cases where the titer decreased from pre- to post-vaccination, a ΔGMT = 0 was used in the calculation of GMR.

### Malaria serology

*P. falciparum *IgG antibodies were measured by the Immunofluorescence Assay (IFA) [37] and by the Enzyme-Linked Immunosorbent Assay (ELISA) [38] following collection of blood 7 months after the children were CH+ or CH- status.

### Statistical Analysis

Sample size was determined initially by a method comparing two proportions. For measles, 215 subjects were required for each group in order to have 90% assurance of significant results to detect this 10% difference in response rates. Similarly, for DTP vaccines, assuming 70% seroconversion for the test group and 60% for the control group, 387 subjects were needed for each group. SAS software, version 9.00 (SAS Institute, USA), was used. Weight-for-height Z-score was calculated using an anthro-system (version 1.02, WHO-CDC, Switzerland).

The primary outcome was rate of seroconversion or seroresponse in CH+ and CH- individuals. As secondary outcomes, geometric mean titers (GMT) and mean fold rise in titer (GMR) were measured for measles, DTP, and malaria antibodies for CH+ and CH- individuals for the strict compliance group.

Study population characteristics at vaccination were compared for the CH+ and CH- children using the Chi-squared test and Student's pooled *t*-test. The Chi-squared test was used for comparison of seroconversion to measles vaccine and seroresponse to DTP vaccinations; the Student's pooled *t*-test was used for pre- and post-vaccination GMT and GMR. A univariate logistic analysis was performed to assess effects of sex, age (> or < 24 months), prophylaxis, weight-for-height Z-score (> or < median Z-score), and pre-vaccination GMT (> or < median GMT) on seroconversion or seroresponse. Multivariate logistic regression was performed on those factors found to be independently associated with seroresponse. Analyses for seroconversion, GMTs, GMRs, and logistic regression were adjusted for village effect.

### Consent

The study protocol was approved by the Burkina Faso (Upper Volta) Ministry of Health and the Institutional Review Board at the CDC. Verbal permission for the study was obtained from the village chiefs, their "council of elders," and each participating family, as was the custom for working in Burkinabe villages.

## Results

The groups were similar at baseline with regard to age, sex, and nutritional status, except for a slight excess of males in the CH- group for DTP (Table 1). Twenty percent of the children were <12 months of age (N = 202). Figure 1 shows the distribution flow of children receiving or not receiving chemoprophylaxis by vaccine type. Although the vaccine was administered as combined DTP, the number of children with serological data that were evaluated differed for the three DTP assays as defined above. The final number of children analysed reflects the availability of paired sera, or loss due to moves or death (Figure 1). When comparing the compliant CH+ children to the non-compliant CH+ children, there were no significant differences in sex, age, or weight-for-height Z-score for those children receiving measles vaccine (P = 0.33, 0.56, 0.52 respectively) or in sex for children receiving DTP (P = 0.22). For the DTP group, age and weight-for-height Z-score was significantly less in the noncompliant CH+ group (P < 0.01, P = 0.02, respectively).

**Table 1:** Characteristics of children qualifying for analyses at vaccination by vaccine type, gender, age and nutrition (measured by the Weight-for-Height Z-score at vaccination) for the prophylaxis (CH+) and no prophylaxis (CH-) groups.

		**Prophylaxis (CH+)**		**No Prophylaxis (CH-)**		
		
**Vacci ne Type**	**Trait**	**#**	% or Mean (SD*)	**#**	% or Mean (SD*)	**P-value**
**Measles**	**Sex % male**	177	53	274	54	0.82
	**Age in months**	178	33.7 (15.2)	274	33.7 (18.1)	0.97
	**Wt-for-Ht Z-score**	162	-0.65 (0.92)	247	-0.54 (1.01)	0.28

**DTP**	**Sex % male**	310	48	204	53	0.04
	**Age in months**	309	32.3 (14.9)	204	32 (17.7)	0.29
	**Wt-for-Ht Z-score**	210	-0.88 (0.98)	37	-0.78 (0.84)	0.29

* SD Standard Deviation

Table 2 shows that seroconversion rates to measles vaccine and seroresponse to diphtheria and tetanus vaccines were not significantly different in the CH+ and CH- groups, both when all children were included and when non-compliant CH+ children were excluded from the analysis (P > 0.05). When all children were included in this analysis, there was a lower rate of seroconversion to diphtheria and tetanus in the CH+ group, but this difference was not statistically significant. Percent seroresponse to pertussis was greater in the CH- group (P < 0.01). In this cluster analysis, there was adjustment for the random effect of village. When the analysis was done without adjusting for the random effect of village, there was a significantly greater rate of seroconversion to diphtheria and tetanus in the CH- group. When non-compliant children were excluded, the difference was no longer significant for diphtheria and tetanus. Percent seroresponse to pertussis remained greater in the CH- group (P < 0.01).

**Table 2:** Seroconversion to measles, diphtheria, tetanus, and pertussis vaccinations in the prophylaxis (CH+) and no prophylaxis (CH-) groups

**Vaccine**	**Proportion With Seroconversion or Seroresponse (%)^†^**		**P-value ** **(Adjusted For Village)**	**Relative Risk (95% CI)**
			
	**CH+**	**CH-**		
**Measles**	127/137(93)*	180/187 (96)	0.16*	0.96 (0.91–1.02)*
	109/116 (94)		0.36	0.98 (0.92–1.03)
**Diphtheria**	108/147(73)*	116/135 (86)	0.26*	0.86 (0.76–0.96)*^‡^
	38/ 46 (83)		0.59	0.96 (0.83–1.12)
**Tetanus**	104/134(77)*	126/138 (91)	0.08*	0.85 (0.77–0.94)*^‡^
	41/43 (95)		0.39	1.04 (0.96–1.14)
**Pertussis**	63/148(43) *	113/168 (67)	<0.01*	0.63 (0.51–0.78)*
	17/44 (39)		<0.01	0.57 (0.39–0.85)

* top rows include all CH+ children (bottom rows include only those CH+ children who met criteria for strict compliance)

† seroconversion is any increase in titer from a negative baseline for measles; seroresponse is a four-fold or greater rise in titer for diphtheria, tetanus, and pertussis

‡ the discordance between the P-value and confidence interval arises because the latter was calculated from a separate analysis which did not adjust for the random effect of village

**Figure 2:**
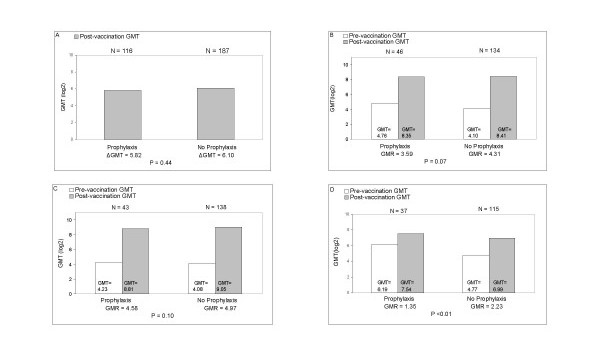
**Pre and post-vaccination Geometric Mean Titers (GMTs) and Geometric Mean Fold Rise (GMR) for prophylaxis (CH+) and no prophylaxis (CH-) groups**. P values listed correspond to the difference in GMR between the two groups. GMTs expressed as log_2_. Children included in the CH+ group met criteria for compliance for chemoprophylaxis. (A) Measles vaccine: GMR for CH+ and CH- groups does not differ significantly. (B) Diphtheria vaccine: GMR for CH+ and CH- groups does not differ significantly. (C) Tetanus vaccine: GMR for CH+ and CH- groups does not differ significantly. (D) Pertussis vaccine: GMR is significantly > for CH- group.

For measles, pre-vaccination measles titers for all children were <1:10 (lowest detectable titer) (Figure 2); GMR was not significantly different in the CH+ vs. CH- group (P = 0.44). For all three antigens GMR was higher in the CH- group, but this difference was statistically significant only for pertussis (P < 0.01).

Multivariate logistic regression analysis indicated that for tetanus, a lower pre-vaccination GMT was positively associated with seroresponse (P = 0.02); for pertussis, a lower pre-vaccination GMT (P < 0.01) and younger age (P = 0.04) were positively associated with seroresponse. There was no significant difference in pre-vaccination pertussis titres between the CH+ and CH- group (P 0.22) when looking at all children regardless of compliance; however, when excluding non-compliant children, pre-vaccination pertussis titres were higher in CH+ children (P <0.01). While pre-vaccination pertussis titres were higher in compliant CH+ children (log_2 _of GMT = 6.19, N = 37) compared to non-compliant CH+ children (log_2 _of GMT = 5.80, N = 84), this difference was not significantly different (P = 0.80). Chemoprophylaxis was not associated with seroresponse for any of the vaccines. Thus, especially for pertussis, the lower vaccine response rate observed in the CH+ group appears to be due, in part, to the greater proportion of subjects with high pre-immunization titers.

Malaria antibody titers were significantly lower in the CH+ group compared to the CH- group following seven months of prophylaxis. GMTs for children receiving measles vaccine were: CH+, 196 (N = 128) vs. CH-, 1089 (N = 219) (P < 0.01) using IFA and CH+, 285 (N = 60) vs CH-, 1990 (N = 64) using ELISA (P < 0.01) and for children receiving DTP vaccine: CH+, 109 (N = 132) vs CH-, 178 (N = 178) (P < 0.01) using IFA and CH+, 86 (N = 63) vs CH-,153 (N = 30) (P = 0.01) using ELISA. Only 13 percent of all CH+ children with detectable malaria titers prior to chemoprophylaxis had undetectable titers post-chemoprophylaxis (N = 159). This indicates that chemoprophylaxis given to young children for five to seven months after previous exposure to malaria was not adequate to eliminate malaria antibodies.

No adverse events were recorded after chemoprophylaxis, blood sample collection, or vaccination other than a few children with 1–3 mm nodules on their arms after receiving the vaccine by injector and one child who developed a cellulitis where the venopuncture occurred: this child recovered with systemic antibiotic treatment.

## Discussion

Proposed mechanisms for malaria-associated immunodepression include impaired macrophage function [39-41], altered cytokine production [39,42], a depletion of T or B cells [43], impaired dendritic cells[42,44,45], elevated nitric oxide production [46] and elevated prostaglandin E during febrile malaria[47]. Clinical evidence includes an association of malaria with increased susceptibility to bacterial infections [48], reactivation of viral infections [49,50], a low prevalence of autoimmune disease in endemic areas[51,52], and reports of decreased responses to vaccinations.

In contrast to asymptomatic parasitaemia, acute malaria impairs vaccine response[8-10,12,17,18,20]. In vitro challenge studies in individuals with acute malaria have demonstrated a depression in the cellular immune response involving alterations in lymphoproliferation and cytokine responses [42,53,54]. The pyrogenic cytokine TNF-alpha is elevated in febrile malaria and may depress humoral immunity by impairing antigen handling by dendritic cells. T-cell levels, CD4 cells in particular, are depressed [55]. IL-1, in addition to TNF-alpha, is elevated in acute illnesses [56,57]. Both promote increased T-cell adhesion to endothelium, which may lead to T-cell margination and sequestration and, thus, a decrease of functional T-cells [55]. CD4 cells secrete cytokines that activate CD8 cells, B-cells and macrophages. In acute malaria, a depression of CD4 cells leads to depressed cellular and humoral immunity, impairing vaccine response.

The absence of association between malaria chemoprophylaxis and vaccine response in this study is consistent with findings from other chemoprophylaxis studies in malarious areas involving children with asymptomatic parasitaemia [4,5,14,16-18,58]. No prior studies have published data on the association between chemoprophylaxis and pertussis (killed, whole-cell) vaccine response in humans.

The agglutination test remains the test of choice for whole-cell pertussis vaccines. Although the antigen-specific ELISA tests can amplify the information provided by the agglutination test, the agglutination test is the only one that has been shown clinically to correlate with vaccine efficacy of whole-cell pertussis vaccines and has been used in relatively recent trials [59,60]. Although ELISA or cell-culture based methods are more widely used today than the passive haemagglutination method for tetanus and diphtheria antitoxins, passive haemagglutination remains acceptable for evaluations of immunized populations [61]. Had they been available, the newer serological tests for measles, including neutralization testing, would have provided greater insight regarding clinical protection from disease.

Results of this study indicated that for the pertussis component, children <24 months of age had a better seroresponse. Vaccinating children <24 months of age will more effectively target the population in greatest need. Pertussis is most serious for very young infants and because complications leading to hospitalization, pneumonias, and death occur most often in those <24 months of age, the recommended age for initiation of pertussis immunization is generally two to three months.

Three doses of DTP vaccine comprise the usual primary series;thus, it would have been useful to assess seroconversion after a third DTP dose in addition to the response following the second dose. Technical and logistical considerations precluded this; additionally, there was concern regarding the possibility of decreased compliance with a longer study, as well as the potential to minimize any differences in the effect of chemoprophylaxis on seroconversion.

Malaria serologies demonstrated a significant difference in GMTs between the two treatment groups at the time of vaccination, adding some assurance that chemoprophylaxis decreased asymptomatic parasitaemia. Despite assumed effective chemoprophylaxis for five to seven months, virtually all compliant children had malaria antibodies; this probably reflected a durable antibody response to infections acquired before the study began. While not the primary study objective, fever prevalence data and blood smear records would have provided valuable insight on malaria prevention in the chemoprophylaxis group. Given efforts to administer intermittent preventive therapy of infants (IPTi) in conjunction with the vaccines included in the EPI, additional prospective studies are needed to establish more firmly the effect of antimalarials on response to childhood vaccinations [6].

## Conclusion

Malaria chemoprophylaxis does not appear to enhance nor impair the antibody response to DTP and measles vaccines. There have been several changes over the 30 years since the study completion, including development of falciparum malaria resistance to 4-aminoquinolones throughout Africa, and establishment of the EPI (1977) and the Roll Back Malaria Partnership (1998). The continuing development and deployment of new antimalarial drugs and childhood vaccines mandates that the possible immunologic and protective interrelationships of these new products be investigated. Studies are in progress by the IPTi Consortium to address these issues  [7].

## Authors' contributions

Dr. Breman was responsible for writing the protocol, carrying out the study in the field, data analysis and writing; Dr. Rosen for synthesis, data analysis and writing; Dr. Manclark for coordinating DTP serologies and doing the pertussis antibody tests; Dr. Meade for editing, assisting with analysis and interpretation of serologic data; Dr. Collins for supervising the malaria antibody testing; Dr. Lobel for assisting with the protocol, field work and expedition of the serologic analyses; Dr. Saliou for participating in field work and manuscript analysis; Ms. Roberts for data registration and preliminary analysis; Dr. Campaoré for serving as the responsible health officer in Burkina Faso; and Dr. Miller for analysis and interpretation of results.

## Financial Support

The study was supported partly by a grant from the Malaria Unit at the World Health Organization. Amodiaquine was contributed by Warner Lambert Pharmaceuticals.
